# Closed genome sequences of *Staphylococcus lloydii* sp. nov. and *Staphylococcus durrellii* sp. nov. isolated from captive fruit bats (*Pteropus livingstonii*)

**DOI:** 10.1099/ijsem.0.004715

**Published:** 2021-03-18

**Authors:** Kay Fountain, Marjorie J. Gibbon, Anette Loeffler, Edward J. Feil

**Affiliations:** ^1^​ Department of Biology and Biochemistry, University of Bath, Claverton Down, Bath, BA2 7AY, UK; ^2^​ Department of Clinical Science and Services, Royal Veterinary College, Hatfield, North Mymms, Hertfordshire, AL9 7TA, UK

**Keywords:** Livingstone’s bats, *Staphylococcus kloosii*, *Staphylococcus durrellii*, *Staphylococcus lloydii*

## Abstract

The increasing availability of whole genome sequencing of bacteria has accelerated the discovery of novel species which may not have been easy to discriminate using standard phenotypic or single gene methods. Phylogenomic analysis of genome sequences from a collection of coagulase-negative staphylococcal species isolated from captive fruit bats revealed two clusters which were close to *Staphylococcus kloosii*. To assess the relatedness of the strains we used digital DNA–DNA hybridization (dDDH) and two methods for average nucleotide identity (ANI) computation which predicted two novel species having dDDH less than 70 % and ANI less than 95%. We propose these species as *Staphylococcus lloydii* sp. nov. (type strain 23_2_7_LY^T^=NCTC 14453^T^=DSM 111639^T^) and *Staphylococcus durrellii* sp. nov (type strain 27_4_6_LY^T^=NCTC 14454^T^=DSM 111640^T^).

Staphylococci inhabit the skin and mucosae of most mammals and birds [1]. They are typically harmless commensal organisms, but can cause bacterial infection as opportunistic invaders of wounds or implant devices, or where host defences are compromised [2]. There are currently more than 50 recognized coagulase-negative and coagulase-positive species within the genus *Staphylococcus,* many of which are difficult to differentiate using either traditional phenotypic methods or single gene methods such as 16S rRNA [3]. Multilocus sequence analysis methods such as ribosomal MLST are more discriminatory, but the increasing availability of whole genome sequencing (WGS) and the use of phylogenomics not only allow the inference of species delineation but also allow species to be placed in their evolutionary context within genera [4–7]. The traditional technique of DNA–DNA hybridization (DDH) has been the gold standard for species delineation of closely related microbial species, but has been mostly superseded by *in silico* methods based on whole genome sequences [6, 7]. The similarity between two whole genomes can be measured as the average nucleotide identity (ANI), with 95 % similarity being a widely accepted species cut-off that is broadly in line with the DDH threshold [8]. Calculation of the genome-to-genome distance (GGD) gives better concurrence with DDH and several methods have been described to infer a digital DDH (dDDH) value based on a 70 % threshold for species identity [6, 7]. Genome blast distance phylogeny (GBDP) is one such method which has demonstrated robust agreement with DDH values, and includes resampling to provide confidence intervals for the results. It is incorporated into the workflow of the Type Strain Genome Server (TYGS), which calculates the closest related type strains, utilizes GBDP to calculate the dDDH, and the intergenomic distances to infer a phylogenetic tree [7].

Whole genome phylogenies of staphylococcal species have been used to define clades within the genus. For example, Naushad *et al.* defined five clades in bovine coagulase-negative staphylococci, the most recently diverged of which contains nine species including *Staphylococcus saprophyticus*, *Staphylococcus arlettae* and *Staphylococcus kloosii* [9].

We have previously assembled a collection of staphylococcal isolates recovered from captive Livingstone’s bats (*Pteropus livingstonii*), a critically endangered species of fruit bat native to the Comoros Islands, and from captive and free-ranging UK native bats [10]. Phenotypic identification of these isolates suggested that *S. kloosii* were numerous, but for some isolates recovered from Livingstone’s bats in Jersey Zoo, the species identification was not supported by matrix-assisted laser desorption-ionization time-of-flight mass spectrometry (MALDI-TOF MS). To help resolve these discrepancies we sequenced the whole genomes of seven representative presumptive *S. kloosii* isolates on the Illumina platform. Phylogenomic analysis of these data revealed two novel lineages related to, but distinct from, *S. kloosii*. We then used long-read sequencing on the Oxford nanopore platform to construct closed hybrid assemblies for a single representative isolate of each of these two clusters, plus a single *S. kloosi* isolate. Calculation of GGD and ANI based on these sequences established that the novel lineages correspond to new species which we designate *Staphylococcus lloydii* sp. nov. and *Staphylococcus durrellii* sp. nov.

## Methods

### Bacterial isolates

Frozen stored isolates from captive Livingstone’s fruit bats in Jersey Zoo (Channel Islands) collected as described in Fountain *et al.*, and from captive and free-ranging UK native bats were used [10]. Isolates had previously been identified phenotypically using the Staph ID32 test kit (bioMérieux) and two MALDI-TOF MS runs, with the second run using an updated database to include more relevant animal associated species (Bruker Microflex LT, Bruker Daltonics; database versions 6 and 7). The identification and origin of the isolates are listed in Table 1.

**Table 1.** Identity and origin of seven presumptive *S. kloosii* isolates from captive bats and results of whole genome sequencing

Isolate ID	Species	Host species (sample site)†	No. of contigs	G+C content %	Length (bp)	Plasmids (bp)
17942E* (2_7_1_Y)	* S. kloosii *	SB (L)	5	32.79	2 700 772	49440, 25020, 21690, 5440
14_7_6_SW	* S. kloosii *	LFB (S)	10	32.85	2 644 309	None
14_4_1_W	* S. kloosii *	WB (S)	12	32.79	2 743 246	31207, 21157, 3877
18_1_E_LY	* S. lloydii *	LFB (ME)	8	33.24	2 544 180	None
23_2_7_LY^T^*	* S. lloydii *	LFB (S)	1	33.24	2 568 697	None
23_2_20_HW	* S. lloydii *	LFB (S)	9	33.5	2 501 648	None
27_4_7_LY^T^*	* S. durrellii *	LFB (O)	3	32.51	2 633 060	86290, 4692

*Denotes isolates with closed genomes assembled from long and short reads. Length and G+C content of assemblies of genomes of *S. kloosii*, *S. lloydii* sp. nov. and *S. durrellii* sp. nov. from bats in base pairs plus lengths of plasmids inferred using Mob-suite [15].

†LFB, Livingstone’s fruit bat; WB, whiskered bat (*Myotis mystacinus*); SB, serotine bat (*Eptesicus serotinus*); L, lesion; S, skin; O, oropharynx; ME, mouth ejecta.

### Phenotypic identification

Isolates were identified using aerobic growth at 37 °C on Columbia agar with 5 % sheep blood, Gram-staining, slide clumping-factor test and tube coagulase test using rabbit plasma (Pro-Lab Diagnostics), catalase test, modified oxidase test using 1 % Kovacs oxidase reagent (Acros Organics, Thermo Fisher Scientific), DNase agar test, oxacillin-resistance screening agar (ORSAB) and Staph ID32 test kit (all reagents from Oxoid, Thermo Fisher Scientific, unless otherwise stated). Further antimicrobial resistance was assessed by disc diffusion tests on Muller–Hinton agar as described in Fountain *et al.* with the addition of the antibiotic polymyxin B (300 IU) [10].

### Genome sequencing

In order to provide additional evidence concerning the identification of the isolates for which the MALDI-TOF MS data was equivocal, we chose seven isolates for full genome sequencing on the Illumina Hi-Seq platform. Although all seven isolates were characterised as *S. kloosi* on the basis of the phenotypic tests, the MALDI-TOF MS data did not confirm these assignments for four of these isolates.

These seven isolates were genome sequenced using Illumina Hiseq (MicrobesNG). DNA was extracted using incubation with lysostaphin, RNase A and proteinase K, followed by purification using SPRI beads. Libraries were prepared using the Nextera XT Library Prep Kit (Illumina) and sequenced on the Illumina HiSeq using a 250 bp paired-end protocol.

Phylogenetic analysis using a maximum-likelihood tree constructed in RAxML-NG based on core-gene analysis of the Illumina data using Roary revealed two clusters that were related to, but distinct from, *S. kloosi* (data not shown) [11, 12]. In order to further characterize these two clusters, a representative isolate from each was selected for long read sequencing using Oxford Nanopore (Oxford Nanopore Technologies), as well as a third isolate confirmed as *S. kloosi*. Genomic DNA was extracted using the Wizard DNA Extraction Kit (Promega). Libraries were prepared using the Rapid Barcoding Kit and multiplexed samples were sequenced using a R9.4.1 flow cell on a MinION (Oxford Nanopore Technologies). Reads were demultiplexed using Deepbinner followed by hybrid assembly to produce a closed genome using Unicycler [13, 14]. Genomes were annotated using Prokka [15]. Potential plasmid sequences were confirmed using plasmidSPAdes and investigated using Mob-recon from Mob-suite to classify them against the database of known plasmids [16, 17]. Closed genomes were aligned using progressiveMauve [18].

### Species identification

ANI comparisons of the genomes were calculated using both the blast method in Kostas Lab webserver and OrthoANI using usearch in Chunlab webserver [8, 19, 20]. The TYGS was used to calculate GGD and 16S rRNA sequence similarity, using genomes in the public databases as described in Meier-Kolthoff *et al.* [7]. The method is as follows: All pairwise comparisons among the set of genomes were conducted using GBDP and accurate intergenomic distances inferred under the algorithm ‘trimming’ and distance formula d5. One hundred distance replicates were calculated each. dDDH values and confidence intervals were calculated using the recommended settings of the Genome-to-Genome Distance Calculator (GGDC 2.1). The resulting intergenomic distances were used to infer a balanced minimum-evolution tree with branch support via FastME 2.1.4 including SPR postprocessing. Branch support was inferred from 100 pseudo-bootstrap replicates each [7].

## Results and discussion

### Genomes

**Fig. 1.**
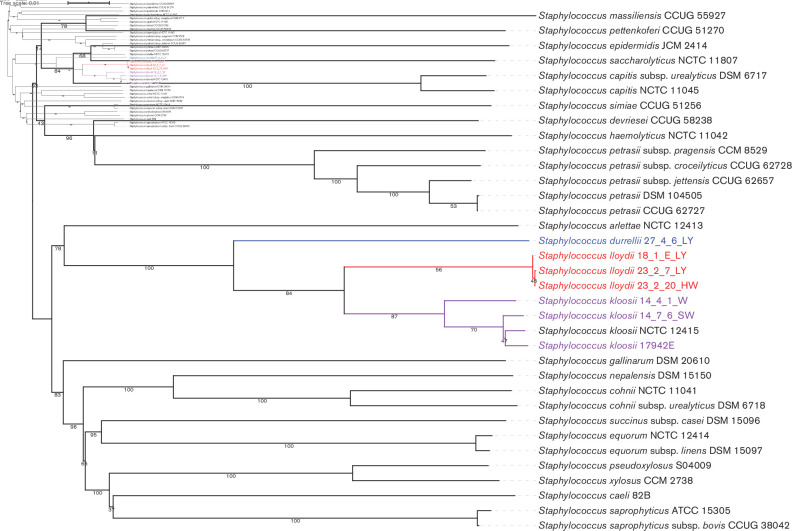
Tree inferred with FastME 2.1.6.1 from GBDP distances calculated from genome sequences using the TYGS workflow [7]. The branch lengths are scaled in terms of GBDP distance formula d5. The numbers above branches are GBDP pseudo-bootstrap support values >60 % from 100 replications, with an average branch support of 77.6 %. The tree was rooted at the midpoint. Coloured branches and tip labels are isolates from bats. Four of these form two distinct clusters separate from any known species (blue and red).

Seven genome sequences were produced: three closed genomes using hybrid assembly of short and long reads, and four more from short reads with fewer than 12 contigs (Table 1). Phylogenetic analysis, ANI and dDDH calculation as detailed below identified two novel species which we henceforth refer to as *S. lloydii* sp. nov. and *S. durrellii* sp. nov. (Fig. 1, Table 2)

**Table 2.** Genome distance measures of seven bat isolates against *S. kloosii* type strain NCTC12415, and mean MALDI-TOF MS score for species assignment ANI>95 %, dDDH>70 % and MALDI-TOF MS>1.7 indicate strong species identification. nr, MALDI-TOF MS score not reported.

Isolate ID	Species	MALDI-TOF MS score (*S. kloosii*)	16S rRNA sequence identity (*S. kloosii* AB009940)	Mean ANI (vs type Strain)	dDDH (vs type strain) [confidence Interval]
17942E (2_7_1_Y)	* S. kloosii *	>2	99.88	99	91.3 [89.1–93.0]
14_7_6_SW	* S. kloosii *	>2	100	99	91.2 [89.0–93.0]
14_4_1_W	* S. kloosii *	>2	100	96	68.4 [65.6–71.4]
18_1_E_LY	* S. lloydii *	1.41	100	90	42.5 [40.0–45.1]
23_2_7_LY^T^	* S. lloydii *	1.47	100	90	42.5 [40.0–45.1]
23_2_20_HW	* S. lloydii *	*S. simiae* (nr)*, S. succinus* (1.31)	100	90	42.4 [39.9–45.0]
27_4_6_LY^T^	* S. durrellii *	1.44	99.53	86	38.1 [35.6–40.6]

### Genome distance analysis

The TYGS analysis identified the 28 type strains that were most similar to the seven genomes from bats. The four bat genomes that were not unequivocally assigned as *S. kloosi* using MALDI-TOF MS are resolved into two novel lineages, one of which is a cluster of three similar isolates, and the fourth (27_4_6_LY^T^) representing a more diverged lineage. These lineages appear distant from all type strains, suggesting they represent two new species. In contrast, the three sequenced isolates that were assigned as *S. kloosi* by MALDI-TOF MS cluster much more closely with the type strain of this species (Fig. 1).

The four isolates corresponding to the two diverged lineages described above have a mean ANI less than 95 % and dDDH less than 70 %, when compared to the *S. kloosi* type strain NCTC 12415 (isolated from squirrel skin), thus confirming they are novel species (Table 2). This analysis also confirms that the other three sequenced isolates that cluster with *S. kloosi* fall within the sequence divergence threshold for this species.

**Fig. 2.**
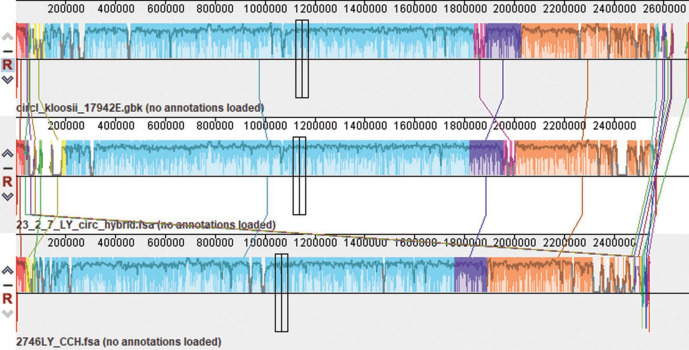
ProgressiveMauve alignment of closed genomes of *S. kloosii*, (top) *S. lloydii* sp. nov. (middle) and *S. durrellii* sp. nov. (bottom) from bats. Apart from one small reordering (magenta) which represents a bacteriophage, there is most variation closest to the origin of replication on the extreme left and right of the figure.

To further compare the genome content of the two novel species with *S. kloosi*, we used progressiveMauve to align the three closed genomes generated using hybrid assembly (Fig. 2). This analysis revealed a high degree of synteny between the genomes (conserved gene order) except for a localized rearrangement (shown in magenta) reflecting bacteriophages present in *S. kloosii* and *S. lloydii* sp. nov. but absent from *S. durrellii* sp. nov. The two phages each have different insertion points and only share around 30 % blastn similarity. All isolates of the two novel species lack the urease operon and phenotypic testing confirmed that they do not produce urease.

*S. durrellii* sp. nov. is predicted by Mob-typer to contain two plasmids classified as novel plasmids closest to pC221 from *S. aureus* (4692 bp) and pC2014-2 from *S. equorum* (86 290 bp) [17].

### Phenotypic and chemotaxonomic characterization

**Fig. 3.**
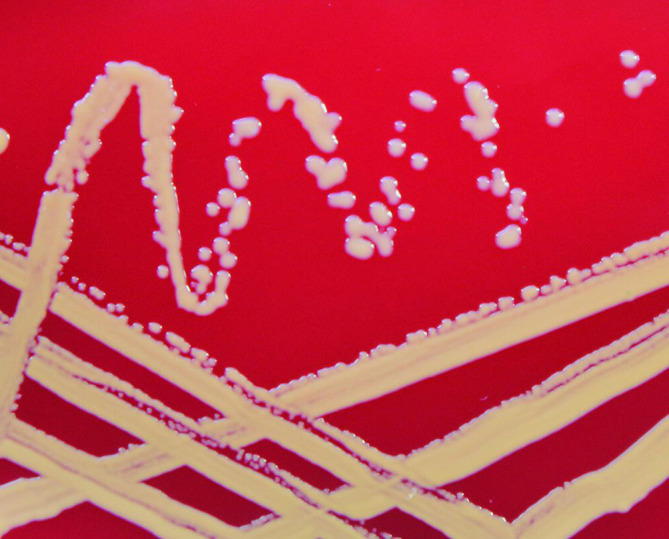
*S. durrellii* sp. nov. (27_4_7_LY^T^) showing yellow pigment and mucoid growth after 24 hours aerobic growth at 37 °C on Columbia agar with 5% sheep blood.

Using Gram stain the isolates were purple cocci, and occurred singly, paired and in clusters. Smooth, shiny, domed, non-haemolytic colonies were seen after 24 h aerobic growth on Columbia 5 % sheep blood agar (Table 3); however, *S. durrellii* sp. nov. also showed mucoid growth (Fig. 3). The phenotypic characteristics of the seven isolates are detailed in Table 4.

**Table 3.** Colony size and colour of seven isolates from bats after 24 h aerobic growth at 37 °C on Columbia agar with 5 % sheep blood

ID	Species	Host species	Colony size	Colony colour
2_7_1_Y (17942E)	* S. kloosii *	Serotine bat	1–2 mm	White/cream
14_7_6_SW	* S. kloosii *	Livingstone’s bat	2–3 mm	White
14_4_1_W	* S. kloosii *	Whiskered bat	1–2 mm	White
23_2_7_LY^T^	* S. lloydii *	Livingstone’s bat	1–2 mm	Yellow/cream
23_2_20_HW	* S. lloydii *	Livingstone’s bat	1–2 mm	White
18_1_E_LY	* S. lloydii *	Livingstone’s bat	2–3 mm	Cream
27_4_7_LY^T^	* S. durrellii *	Livingstone’s bat	1 mm	Yellow

**Table 4.** Phenotypic characteristics of the *S. kloosii* type strain (NCTC 12415) [21], three confirmed *S. kloosii* isolates from bats, three *S. lloydii* sp. nov. isolates from fruit bats and one *S. durrellii* sp. nov. isolate from a fruit bat +, All isolates positive; −, all isolates negative; v, variable (33–66 % of strains positive); nd, not done.

Characteristic	*S. kloosii* type strain (NCTC 12415)	*S. kloosii* (*n*=3)	*S. lloydii* sp. nov. (*n*=3)	*S. durrellii* sp. nov. (*n*=1)
DNase	−	−	−	−
Beta-haemolysis	−	−	−	−
Oxidase	−	−	−	−
Acetoin (Vokes–Proskauer)	−	−	+	−
Urease	+	+	−	−
Arginine dihydrolase	−	−	−	−
Ornithine decarboxylase	−	−	−	−
Aesculin hydrolysis	−	−	−	−
d-Glucose	+	+	+	+
d-Fructose	+	+	+	+
d-Mannose	−	−	−	−
Maltose	+/−	+	+	−
Lactose	+/−	v	−	−
Trehalose	+/−	+	v	+
d-Mannitol	+/−	+	v	+
Raffinose	−	−	−	−
d-Ribose	+	−	−	−
Cellobiose	−	−	−	−
Nitrate reduction	−	−	−	−
Beta-galactosidase	+	+	−	−
Arginine arylamidase	−	v	v	−
Alkaline phosphatase	+	+	v	+
Pyrrolidonyl arylamidase	+	+	+	+
Novobiocin resistance	+	+	+	+
Polymyxin B resistance	nd	−	−	−
Sucrose fermentation	−	−	−	−
*N*-Acetyl-glucosamine fermentation	−	−	v	−
Turanose fermentation	−	−	−	−
l-Arabinose fermentation	+/−	v	−	−
Beta-glucuronidase	+	v	−	+

## Conclusion

We produced whole genome sequence data for staphylococcal isolates that could not be identified with confidence on the basis of MALDI-TOF MS data. Phylogenetic analysis of the genome data revealed two novel clusters, for which a TYGS search did not provide any alternative species designation [6, 7]. Comparisons of representative isolates of these clusters with the *S. kloosi* type strain based on ANI and dDDH confirmed that these isolates were sufficiently divergent as to be regarded as separate novel staphylococcal species which we designate *Staphylococcus lloydii* sp. nov. and *Staphylococcus durrellii* sp. nov. Further work is needed to establish the host species range of these novel species which have so far only been isolated from Livingstone’s fruit bats. The type strains and closed genomes of examples of each species plus *S. kloosii* isolated from bats have been made publicly available.

## Description of *Staphylococcus lloydii* sp. nov.

*Staphylococcus lloydii* (lloy’di.i. N.L. gen. n. *lloydii* after the eminent veterinary dermatologist and microbiologist David H. Lloyd).

Based on the characterization of three isolates originating from Livingstone’s fruit bats the cells are Gram-positive cocci occurring singly, in pairs and clusters. After 24 h aerobic growth at 37 °C on Columbia agar with 5 % sheep blood the colonies are smooth, shiny, circular, domed, white or yellow/cream and 1–2 mm in diameter, displaying no haemolysis. All isolates are catalase-positive, clumping-factor negative, coagulase-negative, oxidase-negative, DNase negative, resistant to novobiocin, sensitive to polymyxin B and grow in 10 % NaCl tryptone soy broth. They are positive for acetoin production by the Vokes−Proskauer reaction and pyrrolidonyl arylamidase. They are negative for urease production, arginine dihydrolase, ornithine decarboxylase, aesculin hydrolysis, nitrate reduction, β-galactosidase and β-glucuronidase. Variable reactions were seen for arginine arylamidase, alkaline phosphatase and *N*-acetyl-glucosamine fermentation. Acid production is positive from d-glucose, d-fructose and maltose; negative from d-mannose, lactose, raffinose, d-ribose, sucrose, turanose, l-arabinose and cellobiose; variable from trehalose and d-mannitol.

The type strain is strain 23_2_7_LY^T^ (NCTC 14453^T^=DSM 111639^T^), isolated in 2015 from the skin of a captive Livingstone’s fruit bat in Jersey Zoo. The genome size is 2 568 697 bp and the DNA G+C content of the type strain is 33.24 mol%.

## Description of *Staphylococcus durrellii* sp. nov.

*Staphylococcus durrellii* (dur.rell’i.i N.L. gen. n. *durrellii* after the innovative naturalist and conservationist Gerald Durrell who founded Jersey Zoo and played a vital role in the establishment of the captive breeding colony of Livingstone’s bats from which the strain was isolated).

Based on the characterization of one isolate originating from a Livingstone’s fruit bat the cells are Gram-positive cocci occurring singly, in pairs and clusters. After 24 h aerobic growth at 37 ℃ on Columbia agar with 5 % sheep blood the colonies are smooth, shiny, circular, domed, yellow and 1–2 mm in diameter, displaying mucoidy but no haemolysis. The isolate is catalase-positive, clumping-factor negative, coagulase-negative, oxidase-negative, DNase negative, resistant to novobiocin, sensitive to polymyxin B and grew in 10 % NaCl tryptone soy broth. It is positive for pyrrolidonyl arylamidase, β-glucuronidase and alkaline phosphatase reactions. It is negative for urease production, acetoin production by the Vokes–Proskauer reaction, arginine dihydrolase, ornithine decarboxylase, aesculin hydrolysis, nitrate reduction, β-galactosidase, arginine arylamidase and *N*-acetyl-glucosamine fermentation. Acid production is positive from d-glucose, d-fructose, trehalose and d-mannitol, and negative from maltose, d-mannose, lactose, raffinose, d-ribose, sucrose, turanose, l-arabinose and cellobiose.

The type strain is strain 27_4_6_LY^T^ (NCTC 14454^T^=DSM 111640^T^) isolated in 2016 from the oropharynx of a captive Livingstone’s fruit bat in Jersey Zoo. The genome size is 2 633 060 bp and the DNA G+C content of the type strain is 32.51 mol%.
